# Machine learning based prediction models for analyzing risk factors in patients with acute abdominal pain: a retrospective study

**DOI:** 10.3389/fmed.2024.1354925

**Published:** 2024-06-05

**Authors:** Tian Gan, Xiaochao Liu, Rong Liu, Jing Huang, Dingxi Liu, Wenfei Tu, Jiao Song, Pengli Cai, Hexiao Shen, Wei Wang

**Affiliations:** ^1^Department of Emergency Medicine, Wuhan Puren Hospital, Wuhan University of Science and Technology, Wuhan, China; ^2^School of Medicine, Wuhan University of Science and Technology, Wuhan, China; ^3^College of Life Science and Technology, Huazhong University of Science and Technology, Wuhan, China; ^4^Maintainbiotech. Ltd. (Wuhan), Wuhan, Hubei, China

**Keywords:** acute abdomen, clinical features, machine learning, artificial neural networks, logistic regression, prediction models

## Abstract

**Background:**

Acute abdominal pain (AAP) is a common symptom presented in the emergency department (ED), and it is crucial to have objective and accurate triage. This study aims to develop a machine learning-based prediction model for AAP triage. The goal is to identify triage indicators for critically ill patients and ensure the prompt availability of diagnostic and treatment resources.

**Methods:**

In this study, we conducted a retrospective analysis of the medical records of patients admitted to the ED of Wuhan Puren Hospital with acute abdominal pain in 2019. To identify high-risk factors, univariate and multivariate logistic regression analyses were used with thirty-one predictor variables. Evaluation of eight machine learning triage prediction models was conducted using both test and validation cohorts to optimize the AAP triage prediction model.

**Results:**

Eleven clinical indicators with statistical significance (*p* < 0.05) were identified, and they were found to be associated with the severity of acute abdominal pain. Among the eight machine learning models constructed from the training and test cohorts, the model based on the artificial neural network (ANN) demonstrated the best performance, achieving an accuracy of 0.9792 and an area under the curve (AUC) of 0.9972. Further optimization results indicate that the AUC value of the ANN model could reach 0.9832 by incorporating only seven variables: history of diabetes, history of stroke, pulse, blood pressure, pale appearance, bowel sounds, and location of the pain.

**Conclusion:**

The ANN model is the most effective in predicting the triage of AAP. Furthermore, when only seven variables are considered, including history of diabetes, etc., the model still shows good predictive performance. This is helpful for the rapid clinical triage of AAP patients and the allocation of medical resources.

## 1 Introduction

AAP is a condition that occurs within the abdomen and has a sudden onset, typically lasting less than a week (1). Patients with AAP are one of the major groups in the ED, accounting for 5–10% of all visits (2–4). AAP can have multiple causes, including gastrointestinal disorders, thoracic cardiovascular disease, and neurological disorders. They also vary in complexity and risk and often involve the clinical care needs of various specialties (including gynecology, pediatrics, internal medicine, surgery, etc.), which may require interdisciplinary and multidisciplinary collaboration, particularly in emergencies such as acute appendicitis, ruptured abdominal aortic aneurysm, and ectopic pregnancy (2, 5, 6). However, in the complex environment of the ED, where most patients with AAP claim to be in urgent need of pain management measures (7, 8), misdiagnosis or other inappropriate management measures can have catastrophic consequences, as well as lead to a range of legal disputes (9–11). This undoubtedly poses a greater challenge to the triage work of healthcare professionals in the ED. Therefore, there is a need to further clarify the risk characteristics of patients with AAP in terms of the severity of their condition. Additionally, there is a need to enhance the ability of healthcare professionals to differentiate the causes of AAP in the ED.

With the utilization of various advanced technologies in clinical medicine, such as computed tomography (CT), ultrasound, and magnetic resonance imaging (MRI), medical imaging plays a crucial role in delivering accurate clinical diagnoses and efficient care for patients with AAP (12, 13). However, non-essential diagnostics inevitably increase the cost of care for patients and the burden on hospital systems. Additionally, there are potential risk factors, such as allergies to contrast media and radiation exposure, that cannot be ignored, particularly in areas with limited healthcare resources (14–16). According to Trentzsch et al., it is crucial to quickly determine the underlying cause in AAP and assess whether urgent or immediate surgical intervention is needed (17). Therefore, there is an urgent need for an efficient triage tool that does not rely solely on radiological techniques but can accurately assess the criticality of AAP based on the physician’s initial assessment and laboratory results. The development of machine learning and artificial intelligence has made this possible. Applying machine learning to emergency triage not only helps improve the accuracy of triage but also reduces the workload of medical staff (18–21). Some studies have attempted to apply machine learning or artificial intelligence to emergency triage of patients with AAP (22–24). However, the clinical indicators included in different studies are often limited by clinical practice experience, and there are also differences in disease assessment standards and hospital preferences for patients seeking treatment. Therefore, the generalization ability of these models in various populations and medical institutions is limited (25, 26). In China, due to the relatively recent implementation of the emergency pre-check triage system, there are limited studies on emergency triage related to AAP, and there is currently no standardized approach. Therefore, to accurately triage patients with AAP and select appropriate treatment strategies, it is crucial to establish a triage prediction model for patients with AAP in our hospital.

In this study, we aimed to construct and optimize a prediction model for AAP triage using a machine learning approach, which is not dependent on imaging diagnosis. To clarify the risky clinical features of AAP patients in terms of the degree of criticality of their condition, so as to achieve accurate triage of patients with AAP.

## 2 Materials and methods

### 2.1 Study design

This retrospective study was conducted in accordance with the Declaration of Helsinki regarding the Ethical Principles for Medical Research Involving Human Subjects (27). The study was approved by the Ethical Committee of Wuhan Puren Hospital (MR-42-24-001914), but informed consent was waived due to the study’s retrospective nature.

### 2.2 Data source

The case information was obtained from the management information system of Wuhan Puren Hospital. The data included the medical records of patients who visited the ED with AAP as their primary complaint between January 1, 2019, and December 31, 2019. A total of 4,323 cases were screened. Epidata version 3.2 was used for data entry. Inclusion criteria: (1) Age ≥ 14 years old; (2) The primary symptom is acute abdominal pain; (3) Complete diagnostic and treatment records with clear diagnosis. Exclusion criteria: (1) Age <14 years old; (2) Patients who did not continue treatment at this hospital and have an unclear diagnosis; (3) Patients who have canceled their appointment; (4) Gynecological acute abdomen, including pelvic inflammatory disease, pelvic mass, torsion of the adnexa, ectopic pregnancy, etc.; (5) Follow-up patients; (6) Patients with more than half of the characteristic variables missing. Based on the nadir criteria, 1911 patient records that did not meet the requirements were removed, and 2,412 patient records that met the criteria were retained for use in this study.

### 2.3 Key feature variables and outcome indicators

The following alternative characteristic variables were identified according to Hastings and Yew et al. (28, 29)

• Patient demographic characteristics: gender, age, profession;
• Past medical history: hypertension, diabetes, coronary heart disease, stroke, history of abdominal diseases;
• Medical visit details: method of visit, time of visit, body temperature, pulse, respiration, blood pressure, blood oxygen saturation;
• General symptoms and signs of the patient: pale appearance, facial appearance, mental status;
• Abdominal characteristics of the patient: bowel sounds, triggering factors, location of the pain, nausea and vomiting, diarrhea and fever, hematemesis and melena, tenesmus, syncope and consciousness disorders, duration of pain, quality of the pain, pain score, rebound tenderness, and abdominal muscle tension.

The outcome indicator was whether the triage level indicated a critical patient. Critical patients were defined as individuals who arrived at the hospital with a report of critical illness within the past 24 h, had a resuscitation record for the first 24 h after arrival, had a critical value recorded within 24 h, required emergency surgery, and ultimately died. If one of these conditions is met, it is coded as 1; otherwise, it is coded as 0.

### 2.4 Data analysis and model construction

Univariate analysis was conducted to screen the characteristic variables. The characteristic variables with a significance level of *p* < 0.05 were selected as independent variables, while the patient’s critical status was used as the dependent variable. Binary logistic regression was used for multifactorial analysis to identify the risk factors associated with the severity of acute abdomen in patients. The characteristic variables identified through logistic regression were then used as input variables for the prediction model. Statistical analyses were performed using SPSS 26.0 software for Windows (SPSS Inc., Chicago, IL, USA). According to the nature of the outcome variable, it can be divided into binary, unordered multicategory, and ordered multicategory data. The chi-square test is used for binary and unordered multicategory data, while Fisher’s exact test is employed when at least one expected frequency is less than five. The Kruskal-Wallis rank sum test is utilized for ordered multicategories. A *p-*value of less than 0.05 was considered statistically significant.

**FIGURE 1:**
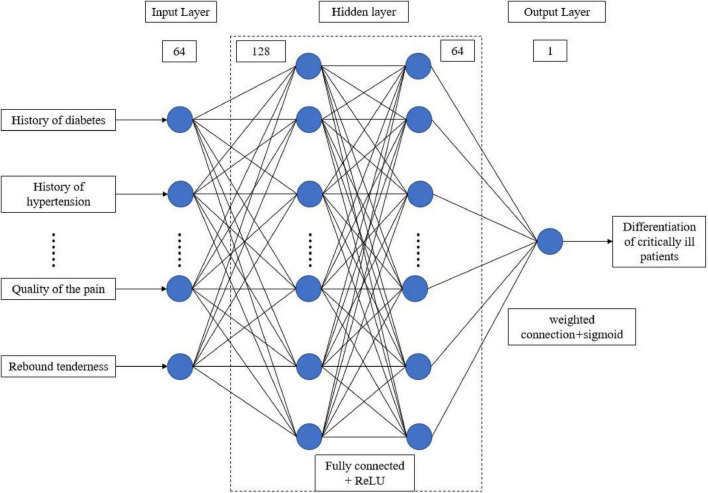
Schematic diagram of the model structure and parameter settings.

A sample database was established based on the statistically significant characteristic variables that were screened. The unbiased randomized sample allocation method was used to code the screened sample data based on the attribute division criteria. The training and testing cohorts were randomly and automatically assigned the data in a 4:1 ratio using Python. A total of eight machine learning models, including logistic regression, K-nearest neighbor, support vector machine, kernel function support vector machine, decision tree, random forest, extreme gradient boosting, and artificial neural network, were built. The evaluation metrics for assessing model performance include accuracy, F1-score, recall, and AUC value. The training, validation, and testing of the model are run based on Python 3.8.8, sklearn 1.3.0, tensorflow-gpu 2.4.0, Keras 2.4.0, with an NVIDIA GeForce 1650 GPU. The structure and parameter settings of the ANN model can be found in Figure 1.

## 3 Results

### 3.1 Characteristics of study subjects

A total of 2,412 cases were included in this study, of which 568 cases (23.5%) were classified as critically ill, and 1,844 cases (76.5%) were not classified as critically ill. There were 1,210 males (50.2%) and 1,202 females (49.8%). The age range of the patients was 15–95 years old. Among them, there were 850 patients aged 15–35 years old, accounting for 35.2% of the total. There were 1,009 patients aged 35–65 years old, accounting for 41.8%. Lastly, there were 553 patients aged 65–95 years old, accounting for 23.0% of the total. Among the patients with different occupations, students accounted for the smallest proportion of 324 cases (13.4%). Most of these cases were non-critical patients (295 cases). Employees made up the highest percentage of patients at 48.5%, with a total of 1,171 cases. Unemployed and retired individuals fell in the middle of the list, with a total of 917 cases. The results of the univariate analysis revealed significant differences between the two groups of critically ill patients and non-critically ill patients in terms of gender, age, and occupation (*p* < 0.05). However, no statistically significant differences were observed in the presence of diarrhea and fever (Table 1).

**TABLE 1:** Demographic characteristics of patients with acute abdomen.

Characteristic variables	Classification	Critically ill (*N* = 568)	Non-critical (*N* = 1844)	Statistical value	*p*
Gender				40.196	<0.001^a^
	Male	351	859		
	Female	217	985		
Profession				250.839	<0.001^a^
	Unemployed or retired	375	542		
	Staff	164	1007		
	Student	29	295		
Age				−16.054	<0.001^c^
	<35	82	768		
	35–65	218	791		
	>65	268	285		
History of hypertension				100.727	<0.001^a^
	Yes	140	161		
	No	428	1683		
History of diabetes				233.399	<0.001^a^
	Yes	99	24		
	No	469	1820		
History of coronary heart disease				151.002	<0.001^a^
	Yes	100	57		
	No	468	1793		
History of stroke				297.946	<0.001^a^
	Yes	96	27		
	No	472	1817		
History of abdominal surgery				297.946	<0.001^a^
	Yes	307	325		
	No	261	1519		
How to come for treatment				255.274	<0.001^a^
	Walk	415	1751		
	Wheelchair	46	5		
	Flatcar	107	88		
Visit time				152.116	<0.001^a^
	0:00—8:00	79	525		
	8:00—17:00	311	502		
	17:00—24:00	178	817		
Temperature				−4.011	<0.001^c^
	Normal	532	1790		
	Low-grade fever	22	42		
	Middle-grade fever	9	7		
	Ardent fever	5	5		
Pulse				−14.453	<0.001^c^
	Overspeed	113	20		
	Normal	442	1824		
	Slow pulse	13	0		
Breathe				−2.797	0.005^c^
	Bradypnea	18	0		
	Normal	549	1838		
	Tachypnea	1	6		
Blood pressure				−16.598	<0.001^c^
	Hypotension	15	0		
	Normal	343	1717		
	Stage 1 hypertension	125	101		
	Stage 2 hypertension	48	24		
	Stage 3 hypertension	37	2		
Degree of blood oxygen saturation				−14.250	<0.001^c^
	Normal	507	1844		
	Decrease	46	0		
	Fulminating anoxia	7	0		
	Critical	8	0		
Pale appearance				1386.94	<0.001^a^
	Yes	420	54		
	No	148	1790		
Facial features				144.546	<0.001^b^
	Normal	131	774		
	Chronic	23	0		
	Acute	409	1070		
	Liver disease related	5	0		
Mind				−9.929	<0.001^c^
	Waking state	538	1844		
	Drowsiness	16	0		
	Lethargic state	5	0		
	Insensible	9	0		
Bowel sounds				−8.528	<0.001^c^
	Weaken	196	5		
	Normal	225	1598		
	Brisk	142	177		
	Hyperfunction	5	64		
Inducement				241.091	<0.001^a^
	No inducement	427	1652		
	Unclean diet	40	156		
	Not easy to digest food	24	6		
	Eat and drink too much	20	6		
	Drink	18	24		
	Other causes, such as the disease	39	0		
Location of the pain				292.386	<0.001^a^
	Middle and upper abdomen	213	612		
	Middle and lower abdomen	25	240		
	Left upper quadrant	41	141		
	Left lower abdomen	7	72		
	Right upper quadrant	7	205		
	Right lower abdomen	82	175		
	Periumbilical	65	108		
	Cartilago ensiformis	57	276		
	Full abdomen	71	15		
Feel sick and vomit				9.375	0.002^a^
	Yes	297	1089		
	No	271	746		
Diarrhea and fever				0.383	0.536^a^
	Yes	72	216		
	No	496	1628		
Vomiting blood and black stool				347.957	<0.001^a^
	Yes	107	6		
	No	461	1838		
Tenesmus				7.29	0.007^a^
	Yes	8	6		
	No	560	1838		
Syncope or a disturbance of consciousness				155.617	<0.001^a^
	Yes	47	0		
	No	521	1844		
Pain duration				42.809	<0.001^a^
	Constant	351	1398		
	Intermittent	217	446		
Quality of the pain				559.907	<0.001^a^
	Hidden pain	251	348		
	Distending	152	1320		
	Dull pain	20	115		
	Colicky	50	51		
	Radiating or spreading	40	5		
	Other	55	5		
Intensity of pain				−10.966	<0.001^c^
	Mild pain	168	1006		
	Moderate pain	371	819		
	Severe pain	29	19		
Tenderness/rebound tenderness				36.128	<0.001^a^
	Yes	447	1204		
	No	121	640		
Abdominal muscle tension				468.384	<0.001^a^
	Yes	192	49		
	No	376	1795		

^a^Chi-square test;

^b^Fisher’s exact test;

^c^Kruskal-Wallis rank sum test.

### 3.2 Multiple logistic regression analysis

The 31 characteristic variables that were found to be statistically significant were included as independent variables in the multifactorial logistic regression analysis. From this analysis, a total of 11 independent risk factors were identified as significantly associated with the dependent variable. These risk factors include the history of diabetes and stroke, pulse, blood pressure, pale appearance, bowel sounds, location of the pain, nausea and vomiting, vomited blood and black stools, quality of the pain, and rebound tenderness (Table 2).

**TABLE 2:** Multiple regression analysis for OR detection.

Characteristic variables	*B*	*SE*	Wald	*p*	OR 95% CI
History of diabetes	3.912	1.524	6.591	0.010	50.006 (2.523–991.144)
History of stroke	2.492	1.246	3.995	0.046	12.080 (1.050–139.017)
**Pulse**					
Overspeed	2.612	1.134	5.305	0.021	13.630 (1.476–125.866)
**Blood pressure**					
Level 1	1.695	0.841	4.062	0.044	5.449 (1.048–28.340)
Level 2	4.504	1.774	6.446	0.011	90.363 (2.792–2924.141)
Level 3	−3.824	1.410	7.355	0.007	0.022 (0.001–0.346)
Pale appearance	6.871	0.908	57.219	0.000	963.943 (162.504–5717.934)
**Bowel sounds**					
Weaken	8.165	1.429	32.641	0.000	3515.393 (213.548–57869.863)
Hyperfunction	−5.186	1.821	8.115	0.004	0.006 (0.000–0.198)
**Location of the pain**					
Right lower abdomen	−1.541	0.730	4.451	0.035	0.214 (0.051–0.896)
Cartilago ensiformis	3.993	1.056	14.295	0.000	54.229 (6.843–429.778)
Nausea and vomiting	−0.918	0.460	3.983	0.046	0.399 (0.162–0.984)
Vomiting blood and black stool	9.246	3.434	7.249	0.007	10359.286 (12.366–8678026.902)
**Quality of the pain**					
Distending pain	−2.413	0.613	15.487	0.000	0.090 (0.027–0.298)
Tenderness/rebound tenderness	1.807	0.803	5.064	0.024	6.091 (1.262–29.388)

B, partial regression coefficient; SE, standard error; OR, odds ratio; CI, confidence interval.

### 3.3 Performance of prediction models

**FIGURE 2:**
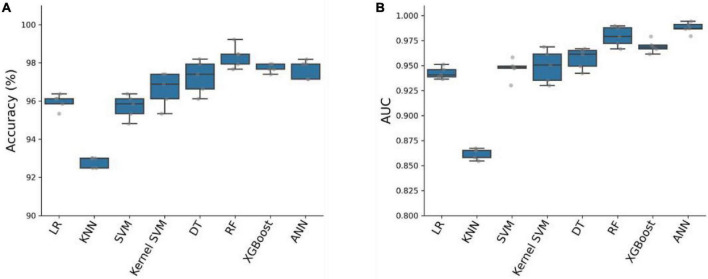
Accuracy **(A)** and AUC **(B)** of eight prediction models on the training cohort with five-fold cross-validation.

To predict critical patients with acute abdomen, we constructed eight models, which can be classified into two categories: (1) Traditional machine learning models: Logistic Regression (LR), K-nearest neighbors algorithm (KNN), Support Vector Machine (SVM), Kernel SVM, Decision Tree (DT), Random Forest (RF), and XGBoost; (2) Artificial Neural Network (ANN). We used 80% of the samples (*n* = 1929) as the training cohort and the remaining 20% (n = 483) as the test set. The remaining 20% of the samples (*n* = 483) serve as the test set. All eight models were first cross-validated on the training set using a 5-fold cross-validation technique. This process was employed to optimize the hyperparameters of the models. The training set was divided into 5 equal parts. Each time, four parts of the data were used as the training cohort, while the predictive performance of the models was evaluated on the remaining part. In total, five training and testing sessions were conducted to determine the optimal hyperparameters for the model. The accuracy and AUC of the eight models were evaluated using five-fold cross-validation, as shown in Figure 2. Among the eight models, the Artificial Neural Network (ANN) model achieved the highest Area Under the Curve (AUC) value of 0.9877 ± 0.0056 and an average accuracy of 97.67% ± 0.48. Although slightly lower than RF and XGBoost, the ANN model performed exceptionally well.

**FIGURE 3:**
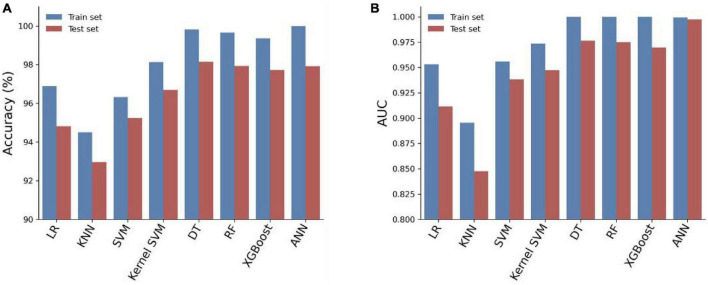
Accuracy **(A)** and AUC **(B)** of eight prediction models on the training and testing cohorts.

We trained eight models using the entire training set and evaluated their predictive performance on the test set. The accuracy, AUC, recall, and F1 scores of the models on the training and test sets are shown in Table 3. Among the eight models, the Artificial Neural Network (ANN) achieved the highest Area Under the Curve (AUC) of 0.9972 on the test set (Figure 3). In addition, the Artificial Neural Network (ANN) achieved an accuracy of 97.92% and an F1 score of 0.9793, which were only slightly lower than the highest performing Decision Tree (DT) model. It is worth noting that the AUC of the ANN on the test set only slightly decreases compared to the AUC on the training set, but it is better than all other models. This indicates that the ANN model has better generalization ability compared to the other models, which is important for clinical applications. Overall, the ANN model has better predictive performance.

**TABLE 3:** Performance comparison of eight models in predicting AAP criticality.

Models	Training cohort evaluation metrics				Test cohort evaluation metrics			
	Accuracy	AUC	Recall rate	F1	Accuracy	AUC	Recall rate	F1
ANN	99.99%	0.9993	(99.80%, 99.57%)	0.9999	97.92%	0.9972	(95.84%, 94.39%)	0.9793
LR	96.89%	0.9531	(98.42%, 92.19%)	0.9689	94.82%	0.9114	(97.17%, 85.11%)	0.9482
KNN	94.50%	0.8953	(99.31%,79.75%)	0.9450	92.96%	0.8474	(98.20%, 71.28%)	0.9296
SVM	96.32%	0.9557	(97.04%, 94.09%)	0.9632	95.24%	0.9382	(96.14%, 91.49%)	0.9524
Kernel SVM	98.13%	0.9734	(98.90%, 95.78%)	0.9813	96.69%	0.9472	(97.94%, 91.49%)	0.9669
DT	99.82%	0.9999	(98.48%, 99.99%)	0.9982	98.14%	0.9763	(98.46%, 96.81%)	0.9814
RF	99.65%	0.9999	(97.86%, 99.99%)	0.9965	97.93%	0.9750	(98.20%, 96.81%)	0.9793
XG Boost	99.36%	0.9999	(97.16%, 99.99%)	0.9936	97.72%	0.9697	(98.20%, 95.74%)	0.9772

### 3.4 Optimization of ANN algorithm prediction model

To further optimize the ANN prediction model, we randomly included a single or a combination of multiple feature variables in the analysis. The results showed that when only a single feature variable was included, the AUC of the ANN model ranged from 0.540 to 0.873. However, when 2–3 variables were included simultaneously, the AUC ranged from 0.599 to 0.905. This suggests that relying solely on clinical feature data of less than 3 variables has significant limitations in AAP triage. In contrast, when we included 11 feature variables obtained from multifactorial logistic regression analyses simultaneously, the AUC reached 0.993. This value was nearly identical to the AUC when all feature variables were included (Table 4). To identify the main factor variables among this group of characteristic variables, various combinations of tests were conducted. The results showed that when including seven variables, history of diabetes and stroke, pulse, blood pressure, pale appearance, and location of the pain, the AUC could reach 0.983. In this situation, we can consider these seven characteristic variables to be significantly important for assessing the severity of AAP.

**TABLE 4:** AUC of the ANN model when incorporating different combinations of feature variables.

Variable combination		AUC
One	History of diabetes	0.8737
	History of stroke	0.5401
	Pulse	0.5763
	Blood pressure	0.6525
	Pale appearance	0.8739
	Bowel sounds	0.8207
	Location of the pain	0.6463
	Nausea and vomiting	0.5729
	Vomiting blood and black stool	0.5781
	Quality of the pain	0.7222
	Tenderness/rebound tenderness	0.6074
Two	History of diabetes and stroke	0.5999
	History of diabetes and pulse	0.6249
	History of diabetes and blood pressure	0.7041
	History of diabetes and pale appearance	0.8955
Three	History of diabetes and stroke, and pulse	0.6524
	History of diabetes, pulse, and blood pressure	0.7224
	History of diabetes, blood pressure, and pale appearance	0.9428
	History of diabetes, pulse, and pale appearance	0.9058
Four	History of diabetes and stroke, pulse, and blood pressure	0.7464
	History of diabetes, pulse, blood pressure, and pale appearance	0.9488
Five	History of diabetes and stroke, pulse, blood pressure, and pale appearance	0.9456
	History of diabetes, pulse, blood pressure, pale appearance, and bowel sounds	0.9699
Six	History of diabetes and stroke, pulse, blood pressure, and pale appearance	0.9704
Seven	History of diabetes and stroke, pulse, blood pressure, pale appearance, and location of the pain	0.9832
Eleven	11 characteristic variables obtained from multivariate logistic regression analysis	0.9932
All	All of the 31 variables	0.9972

## 4 Discussion

Before the widespread availability of medical imaging, the traditional treatment approach depended on the expertise of doctors. They would form their opinions based on the patient’s medical history and physical examination, as well as their own clinical experience (30, 31). In the event of a missed diagnosis or misdiagnosis, it could directly increase the mortality rate (32). To prevent misdiagnosis of critically ill patients with specific conditions, many of them undergo unnecessary surgery (33). Despite the current agreement on the use of CT and ultrasound in AAP, the complexity of AAP still presents a significant challenge for emergency physicians (29, 34–37). Therefore, it is essential to develop a predictive triage system to stratify the risk of AAP patients as accurately as possible (38–40).

In traditional pretest triage, patients are assessed based on age, gender, vital signs, SPO2, consciousness, Glasgow score, blood glucose, and pain (41). However, they are not assessed based on secondary complaints, concomitant symptoms, and past medical history. The advancement of computer-assisted decision-making technology has enabled the assessment of disease risk based solely on clinical information, facilitating rapid triage in the ED. Prediction models exist for common intra-abdominal diseases like appendicitis, pelvic inflammatory disease, and left-sided diverticulitis (42–45). However, there is currently no standardized model for assessing risk and triaging patients with acute abdominal pain (AAP). In this study, we retrospectively analyzed the potential critical risk profiles of patients taking AAP by screening individuals with a comprehensive medical history and clarifying their diagnoses. Our results showed that 11 clinical features, including history of diabetes and stroke, pulse, blood pressure, pale appearance, nausea and vomiting, vomiting of blood and black stools, bowel sounds, location and quality of the pain, and tenderness/rebound tenderness, were strongly correlated with the severity of acute abdomen. The artificial neural network (ANN) model was also effective in predicting the severity of acute abdomen when assessed by combining only seven variables: history of diabetes mellitus, history of stroke, pulse at the time of consultation, blood pressure at the time of consultation, pale appearance, bowel sounds, and site of pain. The three variables of diabetes history, bowel sounds, and pain site were also utilized as key factors in Wang et al.’s risk stratification method for patients with acute appendicitis (46). It is worth mentioning that this method includes more clinical features and laboratory findings. While the method provides an accurate score assessment, it may be somewhat limited in situations where healthcare professionals are initially faced with an urgent claim from an AAP patient. This includes challenges such as the timeliness of laboratory findings and the difficulty in determining the etiology of the disease in some AAP patients, even with laboratory tests (47, 48). In addition, it is important to consider a history of stroke in patients with acute abdominal pain (AAP). These patients may be overlooked during triage because they do not exhibit typical clinical symptoms, despite presenting with abdominal pain. Gastrointestinal symptoms may also trigger central nervous system disorders. For example, Taichi et al. reported a case of acute cerebral infarction caused by colon cancer (49). The determination of the pain site variable aligns with the quadrant partitioning commonly used in most studies (50, 51). Diagnostic imaging and algorithms based on it can facilitate a prompt diagnosis (52, 53). Nevertheless, we need to clarify that in certain special cases, we must consider the potential side effects of radiation exposure, particularly in pregnant patients with AAP (54). However, it is undeniable that laboratory testing and imaging have made an excellent contribution to the management of AAP (55). Sufficient clinical information not only facilitates the interpretation of laboratory results but also helps radiologists make accurate imaging diagnoses (56).

The use of artificial intelligence (AI) in automated agricultural machinery (AAP) can be traced back to the 1970s when it was introduced by Gunn and applied to AAP diagnosis (57). Since then, AI has begun to play a crucial role in the healthcare system and has been consistently optimized. Brejnebøl et al. (58) demonstrated that AI algorithms based on CT scans have benefited the diagnosis of patients with acute appendicitis, albeit with low sensitivity (58). In a recent review, Lam et al. confirmed the significant role of AI in predicting acute appendicitis and emphasized the need for its development in terms of clinical usability (59). It is important to note that artificial intelligence (AI) relies on machine learning, with different algorithmic models producing varying effects. It requires large amounts of data for validation in multiple simulations, making it an exploratory process. For example, three prediction models were developed in a recent study by Henn et al. (60). The tree-based algorithmic model showed the best performance in AAP-assisted decision making. However, even with the incorporation of laboratory test results, its AUC for predicting surgery was only around 0.8. While our model’s performance appears to be strong, it does not necessarily indicate the same level of applicability across different samples. This could be influenced by factors such as sample size, characteristics of the population included, and so on. In addition, it is worth mentioning that AI is not only widely used in the AAP, but also played a crucial role in the 2019 COVID-19 pandemic, helping governments and healthcare workers make timely and accurate judgments, greatly reducing the loss of life and property for people (61).

Although the artificial neural network (ANN) model in this study performs well in predicting APP triage, it still has the following limitations. Firstly, the conclusions drawn from this retrospective study are limited by the available data. Secondly, the time to danger for critically ill patients in this study was defined as 24 h. This definition may focus more on patients with rapidly deteriorating or extremely severe conditions, thereby ignoring those who are equally dangerous but relatively less urgent. It should be noted that due to the limited number of acute abdominal cases related to obstetrics and gynecology in our hospital, it is not representative and therefore excluded from this study. Finally, this was a single-center study, and no additional external validation trials have been conducted.

## 5 Conclusion

Pre-screening and triaging patients with acute abdominal pain is a major challenge in healthcare. In this study, we developed a machine learning algorithm to construct an AAP triage prediction model, with the artificial neural network (ANN) model demonstrating the best performance. The model can assist clinical staff in promptly and accurately identifying patients at high risk of acute abdomen. This enables them to take timely interventions to reduce the danger, relying solely on seven crucial risk factors, especially in situations with limited medical resources. Although an increasing number of studies have begun to focus on the application of AI in clinical diagnostic decision-making, rigorous scientific validation is necessary to assess its clinical usability.

The future emphasis should be on developing and validating joint analysis and prediction models based on multi-center big data. This will help advance the development and application of outcome prediction and treatment plan prediction models.

## Data availability statement

The original contributions presented in this study are included in this article, further inquiries can be directed to the corresponding authors.

## Ethics statement

The studies involving humans were approved by the Ethical Committee of Wuhan Puren Hospital. The studies were conducted in accordance with the local legislation and institutional requirements. The ethics committee/institutional review board waived the requirement of written informed consent for participation from the participants or the participants’ legal guardians/next of kin because This was a large retrospective study.

## Author contributions

TG: Conceptualization, Supervision, Formal analysis, Funding acquisition, Resources, Writing – original draft. XL: Conceptualization, Writing – original draft, Data curation, Investigation, Validation. RL: Data curation, Investigation, Writing – original draft, Formal analysis, Methodology. JH: Data curation, Formal analysis, Investigation, Methodology, Writing – original draft. DL: Data curation, Formal analysis, Methodology, Writing – original draft, Software, Visualization. WT: Data curation, Methodology, Software, Visualization, Writing – original draft. JS: Methodology, Writing – original draft, Formal analysis, Validation. PC: Formal analysis, Methodology, Validation, Writing – original draft. HS: Conceptualization, Project administration, Supervision, Writing – review and editing. WW: Conceptualization, Project administration, Resources, Supervision, Writing – review and editing.
